# Supplementary material for: Combined Effects of Fibroblast Growth Factor-2 and Carbonate Apatite Granules on Periodontal Healing: An In Vivo and In Vitro Study
Source: Biomedicines. 2024 Jul 25;12(8):1664. doi: 10.3390/biomedicines12081664 (PMC11352071; doi:10.3390/biomedicines12081664)
Supplement: Supplementary file 1 [file biomedicines-12-01664-s001.zip › biomedicines-3120284-supplementary.pdf]

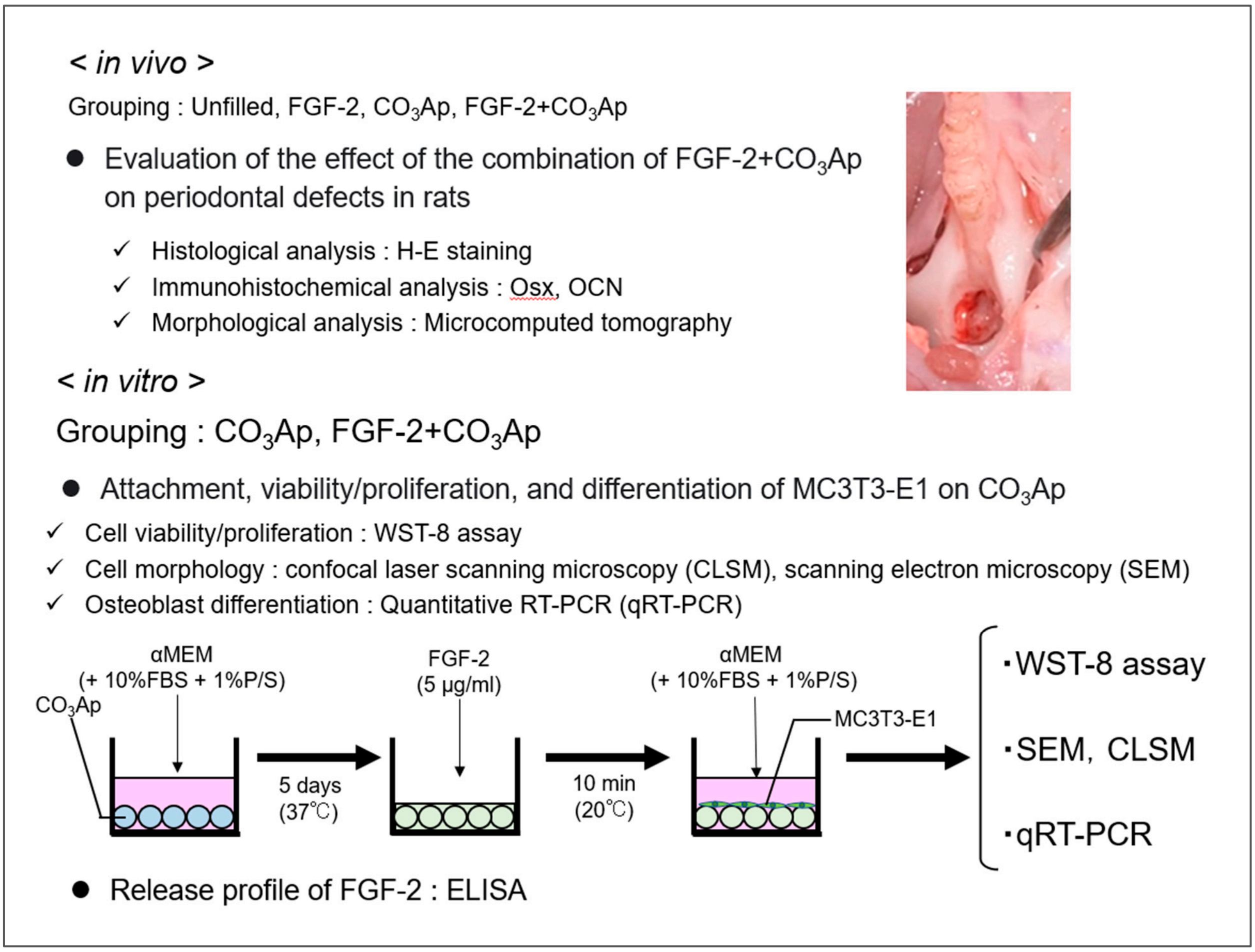


**Figure S1**. **An overview of the experiments**

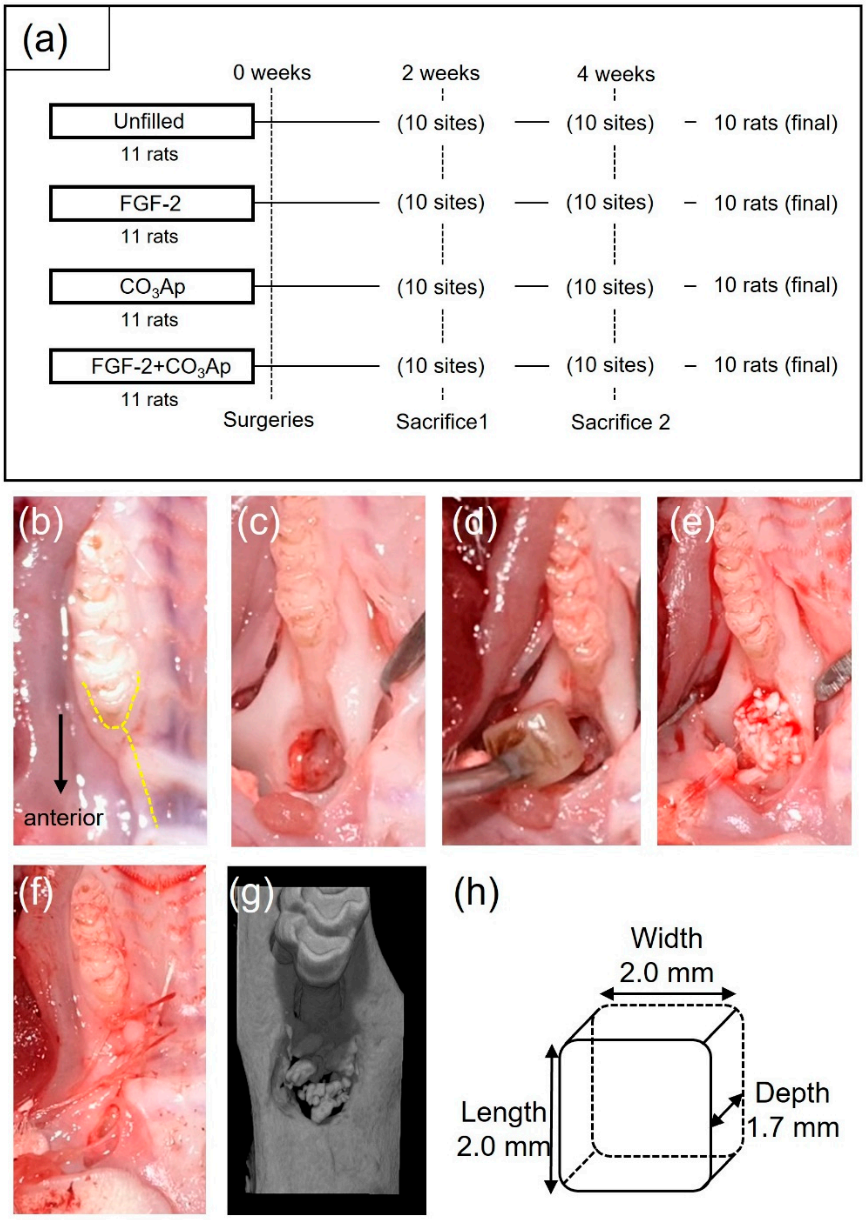


**Figure S2. In vivo protocol and surgical creation of periodontal defects (FGF-2 + $CO_3Ap$ group).**
(a) Experimental protocol and groups (b) Incision design: yellow dotted line (c) After raising full-thickness flaps, bilateral standardized periodontal defects (2.0 × 2.0 × 1.7 mm) were created mesially of the maxillary first molars (M1). (d) Using surgical template. (e) Application of $CO_3Ap$ (pre-mixed with FGF-2). (f) Flaps were closed using resorbable sutures. (g) Micro-CT image of the periodontal defect immediately after surgery. (h) Standardized defect size.

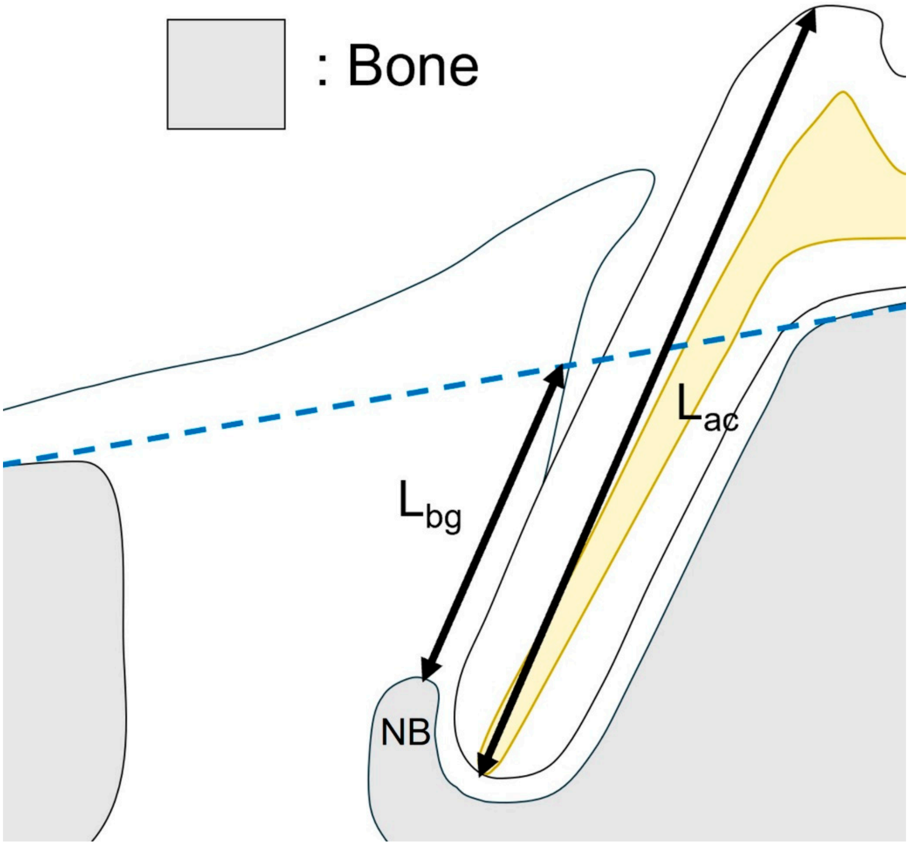


**Figure S3**. **Measurement method of relative new bone height for histomorphometric analysis.**

$L_{ac}$, the length between the apex to cusp of the first molar; blue dotted line = the level of intact alveolar bone; $L_{bg}$, the bone gap; length between the most coronal extent of newly-formed bone and the bule dotted line ($L_{bg}$ is parallel to Lac); NB, new bone. Relative new bone height was determined by ($L_{ac}$) / ($L_{bg}$).

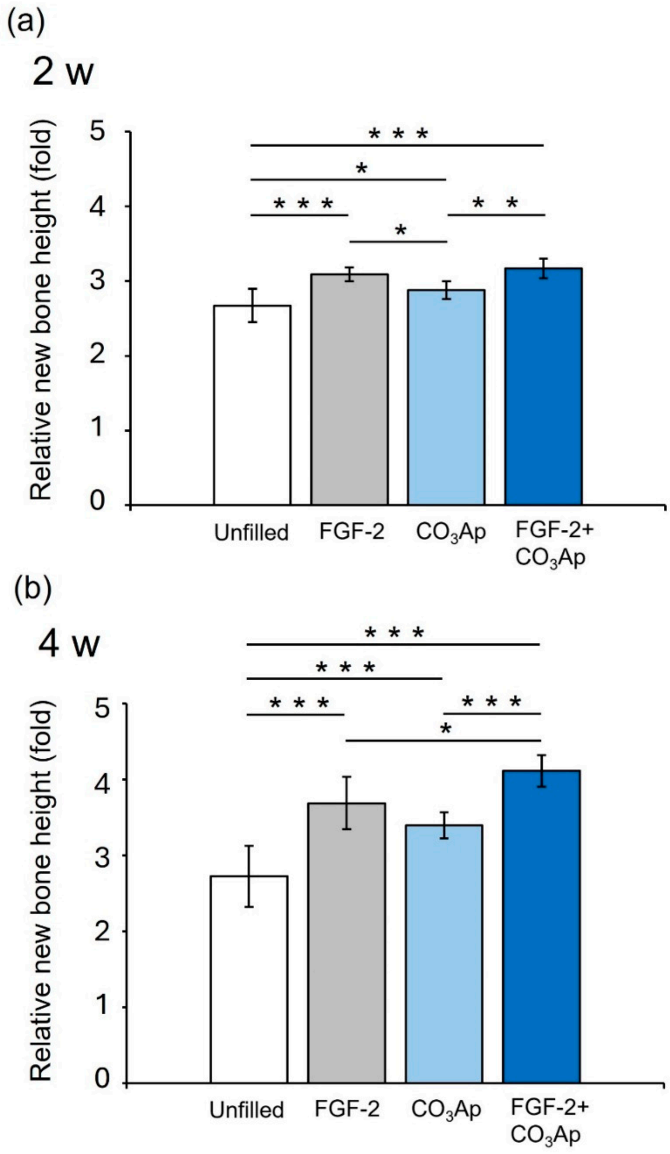


**Figure S4**. **Histomorphometric assessment of relative new bone height.**
(a) Relative level of new bone at 2 weeks. The FGF-2, $CO_3Ap$, and FGF-2 + $CO_3Ap$ groups exhibited significantly higher level than the Unfilled group. The FGF-2 and FGF-2 + $CO_3Ap$ groups showed significantly higher level than the CO3Ap group. (b) Relative level of new bone at 4 weeks. The FGF-2, $CO_3Ap$, and FGF-2 + $CO_3Ap$ groups exhibited significantly higher level than the Unfilled group. The FGF-2 + $CO_3Ap$ group demonstrated significantly higher level compared to the FGF-2 and $CO_3Ap$ groups. Relative level of new bone was compared between groups. Data shown as mean ±SD (*n* = 8). *p < 0.05, **p < 0.01 , ***p < 0.001, by ANOVA with Tukey post hoc test.

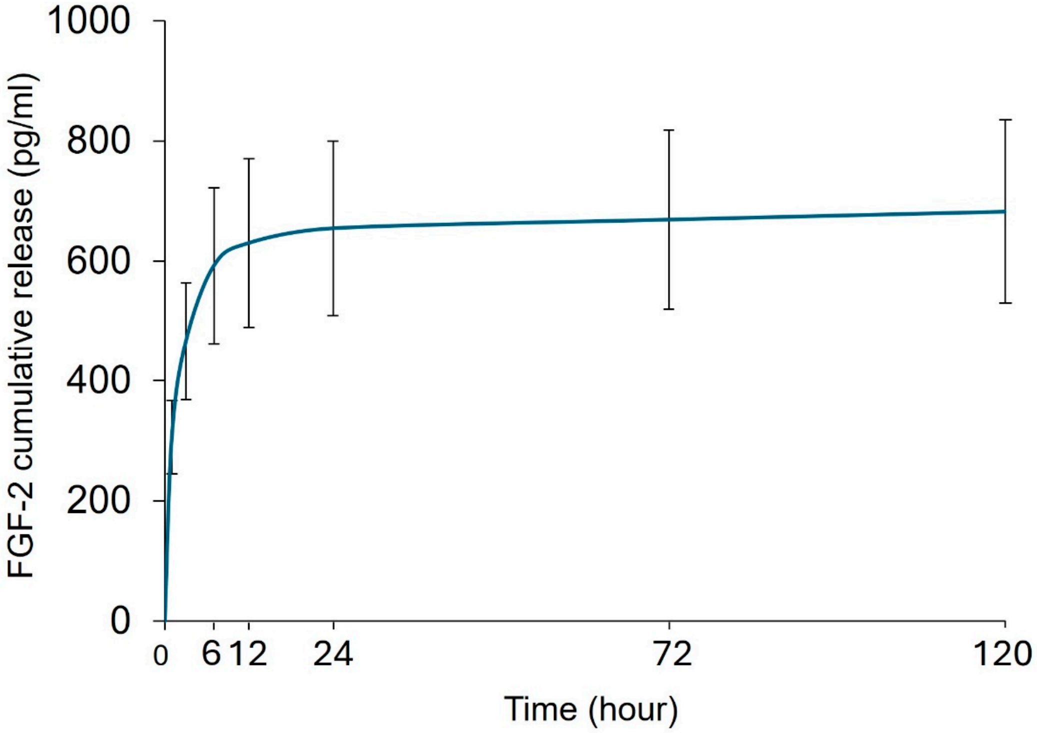


**Figure S5**. **Kinetics of FGF-2 cumulative release from FGF-2-treated $CO_3Ap$.** Kinetics of FGF-2 cumulative release from $CO_3Ap$. FGF-2 release evaluated by ELISA. FGF-2 was released from $CO_3Ap$ treated with FGF-2 over 120 hours. Data shown as mean ± SD ($n = 5$).
